# Supplementary material for: MetaRibo-Seq measures translation in microbiomes
Source: Nat Commun. 2020 Jun 29;11:3268. doi: 10.1038/s41467-020-17081-z (PMC7324362; doi:10.1038/s41467-020-17081-z)
Supplement: Supplementary file 10 — Supplementary Data 7 [file 41467_2020_17081_MOESM10_ESM.zip › File2/Confidence_High_Taxonomy/177841_out.krona.html]

Javascript must be enabled to view this page.

members
magnitude
magnitudeUnassigned
count
unassigned
taxon
rank

177841\_out

142

2
142
superkingdom

1
1239
phylum

class
1
909932

1843488
1
order

1
909930
family

genus
33024
1


SRS048164\_contig\_number\_31286
1
626940
species

976
141
phylum

200643
141
class

141
171549
order

815
2
family

816
2
genus


SRS050998\_contig\_number\_11572
820
1
species


SRS024625\_contig\_number\_1919
329854
1
species

1853231
139
family

139
genus

SRS011302\_contig\_number\_contig-100\_15535.56650SRS012969\_contig\_number\_contig-100\_29664.29664SRS015217\_contig\_number\_contig-100\_25808.74281SRS015960\_contig\_number\_contig-100\_794.112033SRS016495\_contig\_number\_contig-100\_17524.17524SRS017745\_contig\_number\_contig-100\_21636.21637SRS018817\_contig\_number\_15339SRS018984\_contig\_number\_22063SRS019381\_contig\_number\_19346SRS020233\_contig\_number\_54650SRS023583\_contig\_number\_2279SRS045244\_contig\_number\_6973SRS045713\_contig\_number\_16169SRS047044\_contig\_number\_2911SRS053214\_contig\_number\_contig-100\_192.107768SRS075821\_contig\_number\_2134SRS077730\_contig\_number\_contig-100\_312.100938SRS098061\_contig\_number\_contig-100\_20318.58550SRS1054716\_contig\_number\_contig-100\_28189.28190SRS143181\_contig\_number\_contig-100\_37439.37439SRS143372\_contig\_number\_contig-100\_31124.31125SRS143523\_contig\_number\_contig-100\_36.48117SRS145497\_contig\_number\_6250SRS893231\_contig\_number\_contig-100\_17.18SRS893342\_contig\_number\_404
283168
25

species
28118
92

SRS011239\_contig\_number\_19625SRS012849\_contig\_number\_22327SRS013800\_contig\_number\_11207SRS014683\_contig\_number\_13159SRS015782\_contig\_number\_32042SRS015890\_contig\_number\_19686SRS016018\_contig\_number\_11144SRS016056\_contig\_number\_3045SRS016095\_contig\_number\_13089SRS017247\_contig\_number\_3140SRS017521\_contig\_number\_29831SRS017821\_contig\_number\_23255SRS018133\_contig\_number\_11867SRS018623\_contig\_number\_17309SRS018836\_contig\_number\_27087SRS018888\_contig\_number\_contig-100\_862.50418SRS019030\_contig\_number\_15856SRS019068\_contig\_number\_62035SRS019161\_contig\_number\_20943SRS019397\_contig\_number\_2757SRS019445\_contig\_number\_20708SRS019601\_contig\_number\_12937SRS020328\_contig\_number\_13087SRS020394\_contig\_number\_14797SRS021219\_contig\_number\_4003SRS021484\_contig\_number\_16838SRS022137\_contig\_number\_2164SRS024075\_contig\_number\_9818SRS043001\_contig\_number\_2400SRS043841\_contig\_number\_1577SRS045645\_contig\_number\_2196SRS046068\_contig\_number\_45SRS046369\_contig\_number\_9125SRS047433\_contig\_number\_18470SRS048870\_contig\_number\_14411SRS049446\_contig\_number\_10801SRS049773\_contig\_number\_20591SRS049900\_contig\_number\_8233SRS050925\_contig\_number\_18104SRS052697\_contig\_number\_19566SRS053398\_contig\_number\_12662SRS054352\_contig\_number\_contig-100\_27614.65910SRS054905\_contig\_number\_20905SRS055982\_contig\_number\_13725SRS056273\_contig\_number\_24679SRS058770\_contig\_number\_14904SRS063040\_contig\_number\_27432SRS063190\_contig\_number\_10678SRS063370\_contig\_number\_3226SRS063518\_contig\_number\_14971SRS063985\_contig\_number\_25257SRS064276\_contig\_number\_17744SRS064557\_contig\_number\_19140SRS064757\_contig\_number\_contig-100\_1002.123634SRS075773\_contig\_number\_1733SRS077231\_contig\_number\_24339SRS077392\_contig\_number\_27790SRS078242\_contig\_number\_14382SRS097958\_contig\_number\_contig-100\_833.113220SRS098571\_contig\_number\_35353SRS098644\_contig\_number\_36443SRS098881\_contig\_number\_15786SRS100021\_contig\_number\_10667SRS103987\_contig\_number\_18767SRS1041134\_contig\_number\_8505SRS1041137\_contig\_number\_5812SRS104311\_contig\_number\_9222SRS104636\_contig\_number\_17138SRS104975\_contig\_number\_16835SRS105153\_contig\_number\_24295SRS140513\_contig\_number\_10905SRS142503\_contig\_number\_17321SRS143417\_contig\_number\_16231SRS143598\_contig\_number\_13247SRS143876\_contig\_number\_14873SRS143895\_contig\_number\_contig-100\_994.225472SRS144183\_contig\_number\_35587SRS144362\_contig\_number\_24924SRS144506\_contig\_number\_22488SRS146764\_contig\_number\_18910SRS147271\_contig\_number\_23805SRS147346\_contig\_number\_35138SRS148159\_contig\_number\_36172SRS148196\_contig\_number\_42010SRS148424\_contig\_number\_contig-100\_1367.162912SRS148721\_contig\_number\_33089SRS149181\_contig\_number\_27616SRS893252\_contig\_number\_18874SRS893279\_contig\_number\_12713SRS893341\_contig\_number\_5248SRS893369\_contig\_number\_363SRS893378\_contig\_number\_21311


SRS011084\_contig\_number\_25203SRS015431\_contig\_number\_54963SRS024435\_contig\_number\_24SRS044535\_contig\_number\_13082SRS046717\_contig\_number\_6949SRS049402\_contig\_number\_13771SRS051610\_contig\_number\_10830SRS065504\_contig\_number\_12256SRS076804\_contig\_number\_19673SRS140492\_contig\_number\_20835SRS148091\_contig\_number\_5589
11
1897010
species


SRS015264\_contig\_number\_7087SRS050422\_contig\_number\_20912SRS062654\_contig\_number\_21639SRS076976\_contig\_number\_15161SRS077086\_contig\_number\_14553SRS077127\_contig\_number\_14757SRS077552\_contig\_number\_18851SRS101433\_contig\_number\_12916SRS143991\_contig\_number\_26984
9
1263090
species

2
1637502

SRS077294\_contig\_number\_contig-100\_901.94840SRS077502\_contig\_number\_5330
species
